# Backscattering Analysis at ATR on Rough Surfaces by Ground-Based Polarimetric Radar Using Coherent Decomposition

**DOI:** 10.3390/s23073614

**Published:** 2023-03-30

**Authors:** Anton V. Kvasnov

**Affiliations:** School of Cyber Physical Systems and Control, Peter the Great St. Petersburg Polytechnic University, St. Petersburg 195251, Russia; antonkv@mail.ru or kvasnov_av@spbstu.ru

**Keywords:** automatic target recognition (ATR), radar polarimetry, object recognition, polarimetric radar, rough surfaces, high-resolution range profile (HRRP), coherent decomposition

## Abstract

This article deals with the analysis of backscattering at automatic target recognition (ATR) by ground-based radar located on rough terrain surfaces, using the properties of wave polarization. The purpose of the study is to examine and compare linear and circular polarized reflected waves, which can be described by decomposition theorems. Coherent decompositions (Pauli, Krogager, Cameron decomposition) are considered in the case of a rough terrain, for which the advantage of the Pauli decomposition has been shown. The article demonstrates an approach to the extraction of polarization signal backscattering data for two types of vehicles with different profiles. It is shown that the measurement results can be calibrated by a corner reflector that takes into account the properties of the ground surface, and further used for ATR based on supervised learning algorithms. The accuracy of object classification was 68.1% and 54.2% for the signal generated by linearly and elliptically polarized waves, respectively. Based on these results, we recommend using a linearly polarized wave as an object recognition mechanism. At the same time, any reflected depolarized wave significantly reshapes the structure due to the rotation of the object profile and the influence of a rough surface (vegetation fluctuations). This explains the low recognition accuracy in general.

## 1. Introduction

**Figure 1:**
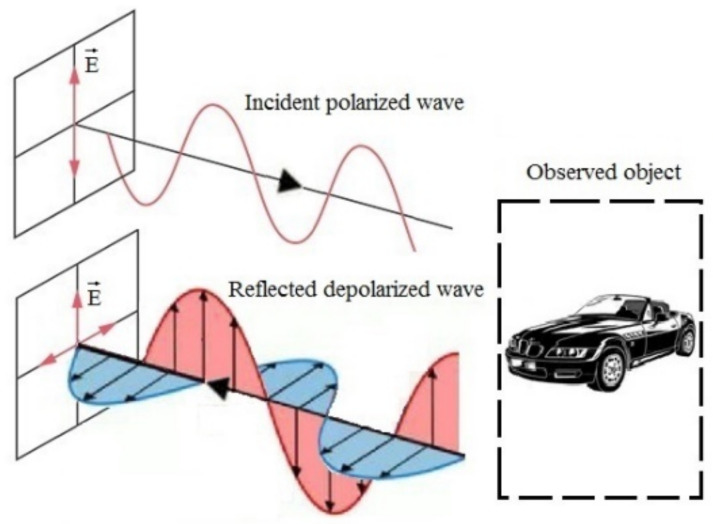
Principle of obtaining a polarized target signature. E—vector of electric-field intensity.

The problem of scattered polarized electromagnetic wave processing on arbitrary objects has many applications. In particular, the analysis of polarization characteristics enables ATR using supervised learning algorithms [1,2]. Having obtained a stable scattering pattern using radar polarimetry, we can use these data to classify a remotely sensed object, as denoted in [2,3,4]. It is known that polarimetric radar is capable of emitting and receiving signal with linear and circular polarization reflected from objects of various types and shapes (Figure 1). Although such properties allow to extend the invariant feature space for ATR, we face a number of problems in signal processing. Thus, let us trace the retrospective of radar polarimetry and its applications.

The basic principles of polarization scattering were initiated in [5,6]. The use of polarization properties of the radar was continued in [7,8]. Theorems on decomposition of the target into orthogonal components have become important achievements [9,10]. Currently, an important task of radar polarimetry is the interpretation of target signatures and their application in various practical developments [11,12,13,14].

Polarimetric radar for object recognition offers broad opportunities in meteorological observations [15,16,17], classification of birds and insects [18,19], and in automatic MIMO applications [20,21,22]. A large class of practical recognition problems based on radar polarimetry relates to ground-based space surveys in synthetic aperture radar (SAR) [22,23,24]. Therefore, a special mathematical approach using matrix cells has been developed [7,25]. Another method of ATR is the use of high-resolution range profile (HRRP) in radar systems [26,27]. At the same time, there are several studies on ATR of polarimetric ground-based radars under different interference conditions on a rough surface [1,2,28].

In this case, the main problem is the influence of HRRP on the accuracy of object classification [29,30,31]. On the other hand, the physical influence of the ground as a polarimetric discriminant for ATR is also essential [1,2,30]. Therefore, this article aims to investigate the implementation of rough terrain object recognition based on a number of backscattering marks received experimentally for emitted linearly and circularly polarized signals.

Note that most of the works did not take into account scattering on rough ground surfaces for a small elevation angle [26,27]. Some of the results were simulated without experimental data [2] or under laboratory conditions [1,22]. In some works, where the classification of objects in polarimetric radar was carried out, the scattering of emitted elliptically polarized waves was not taken into account [21,28].

### 1.1. Problem and Tasks

In this article we want to focus on the problem of the effect of a rough surface on ATR. Based on this problem, the author considers the following tasks:

- Select an object decomposition theorem for feature space design and test an acceptable scattering mechanism after calibration
- Conduct data calibration considering the effect of rough ground surface on object recognition
- Explore supervised learning as an ATR tool for polarimetric radar.

The article evaluates the efficiency of object recognition on real rough surfaces using polarization data obtained with Ku-band radar. In the course of the study, the theoretical results were tested on real data. Thus, the results of HRRP testing are tentatively presented for discussion. 

### 1.2. Structure of the Article

**Figure 2:**
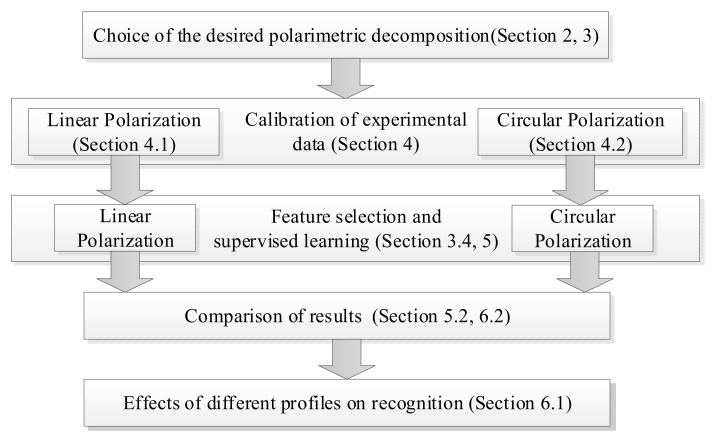
Structure of the article.

Since our study is mainly based on experimental evaluation, the structure of the article is designed according to Figure 2.

In Section 2 we present the necessary theoretical aspects. Section 3 discusses applied polarization characteristics and features that we can obtain for different types of decomposition. Section 4 and Section 5 demonstrate results of the experimental data. Section 6 and Section 7 present the results, discussions, and conclusions of the article. 

## 2. Theoretical Part

In this section, we will present the basic decomposition theorems and make a number of assumptions concerning the conditions of wave propagation along a rough underlying surface. 

### 2.1. Assumptions

The problem of optimal data extraction from reflected signals was posed in the dissertation [5]. In the general case, it is necessary to find a mathematical model in which the target profiles are invariable to changes in the wave polarization basis. One can distinguish four main classes of theorems describing the scattering matrix decomposition [11,12,14]:

- Kennaugh matrix dichotomy (Kennaugh–Huynen scattering matrix)
- Decomposition of the covariance matrix (Freeman and Durden)
- Eigenvector decomposition of the coherent scattering matrix (Cloude–Pottier target scattering decomposition) [32,33]
- Coherent scattering matrix decomposition (Pauli, Krogager, Cameron).

In the general case we consider the situation with a rough ground surface and a complex shape of the object under study. The depolarized signal from the reflecting object has an individual amplitude attenuation and phase shift, which depend on the target profile and wavelength. Let us take into account some assumptions:

1. The transmitted electromagnetic wave is a plane monochromatic wave with constant frequency, amplitude, and initial phase in time.
2. In the propagation of a polarized wave, there are additive and multiplicative interferences.
3. Polarimetric radar generates a signal with strict linear (vertical and horizontal) and strict circular (right and left) polarization.
4. The classification procedure involves obtaining labeled data on detected objects; hence it is a supervised learning task.

The coherent decomposition is performed as a combination of basis matrices corresponding to canonical scattering mechanisms [33]. Any classification requires the use of independent orthogonal features for the training sample. Therefore, the mathematical model must be developed using coherent decompositions of the Pauli, Krogager, or Cameron methods, which are discussed further in Section 3.

### 2.2. Polarized Wave Model 

Before studying the theorems of coherent decompositions, let us turn to the basic techniques of polarimetric data processing. The problem of polarized wave scattering on inhomogeneous objects is solved using two approaches [11].

1. The Jones calculus. This is a mathematical description of a fully polarized wave in which the Jones vectors and linear elements of the Jones matrix (Equation (1)) determine the polarization:
  (1) $$E_{R}=JE_{T}:\;\left|\begin{array}{c} E_{x}^{R}e^{j\phi_{x}^{R}}\\ E_{y}^{R}e^{j\phi_{y}^{R}} \end{array}\right|=\left(\begin{array}{cc} J_{xx}e^{j\phi_{xx}} & J_{yx}e^{j\phi_{yx}}\\ J_{xy}e^{j\phi_{xy}} & J_{yy}e^{j\phi_{yy}} \end{array}\right)\times \left|\begin{array}{c} E_{x}^{T}e^{j\phi_{x}^{T}}\\ E_{y}^{T}e^{j\phi_{y}^{T}} \end{array}\right|$$
  where $E_{x}^{T}e^{j\phi_{x}^{T}},\;E_{x}^{R}e^{j\phi_{x}^{R}}$ are amplitudes and phases of the transmitted and received signals, respectively, along the Ox axis; $E_{y}^{T}e^{j\phi_{y}^{T}},\;E_{y}^{R}e^{j\phi_{y}^{R}}$ are amplitudes and phases of the transmitted and received signals, respectively, along the Oy axis; $J_{xx}e^{j\phi_{xx}},\;J_{yy}e^{j\phi_{yy}}$ are complex reflection coefficients expressing the direct transformations of the incidental wave; and $J_{xy}e^{j\phi_{xy}},\;J_{yx}e^{j\phi_{yx}}$ are complex reflection coefficients expressing the cross-transformations of the incidental wave.
2. Mueller calculus. A mathematical description of arbitrarily polarized scattering is given by the Stokes vector, which is expressed as follows:
  (2) $$S^{R}=MS^{T}:\;\left[\begin{array}{c} s_{0}^{R}\\ s_{1}^{R}\\ s_{2}^{R}\\ s_{3}^{R} \end{array}\right]=\left(\begin{array}{cccc} m_{00} & m_{01} & m_{02} & m_{03}\\ m_{10} & m_{11} & m_{12} & m_{13}\\ m_{20} & m_{21} & m_{22} & m_{23}\\ m_{30} & m_{31} & m_{32} & m_{33} \end{array}\right)\times \left[\begin{array}{c} s_{0}^{T}\\ s_{1}^{T}\\ s_{2}^{T}\\ s_{3}^{T} \end{array}\right]$$
  where $S^{R}={\left[\begin{array}{cccc} s_{0}^{R} & s_{1}^{R} & s_{2}^{R} & s_{3}^{R} \end{array}\right]}^{T}$ denotes the Stokes vector of scattering waves, and $S^{T}={\left[\begin{array}{cccc} s_{0}^{T} & s_{1}^{T} & s_{2}^{T} & s_{3}^{T} \end{array}\right]}^{T}$ denotes the Stokes vector of an incidental wave; and $M\left\{4\times 4\right\}$ is the Muller matrix, which characterizes the scattering properties of the object.

The polarimetric coherency in the monostatic case of backscattering must satisfy the so-called reciprocity and symmetry [11]. Then the Muller matrix can be reduced to the unitary 3 × 3 matrix of the special group $M\left\{4\times 4\right\}\to T_{COH}\left\{3\times 3\right\}$ for the monostatic case. Then the Muller value given is as

(3) $$\langle m_{00}^{2}\rangle =\langle m_{11}^{2}\rangle +\langle m_{22}^{2}\rangle +\langle m_{33}^{2}\rangle$$

where $m_{00},\;m_{11},\;m_{22},\;m_{33}$ denote the main elements of the Muller matrix; and $\langle \;\cdot \;\rangle$ denotes their average value. Several differences between the two approaches are worth noting [11].

1. The Mueller calculus has only a phenomenological interpretation and is not related to the electromagnetic theory, whereas the Jones calculus derives directly from this theory.
2. The Jones calculus allows for the absolute phase, while the Mueller calculus does not consider the phase at all.
3. The elements of the Jones matrix correspond to the radiation amplitude, while the elements of the Muller matrix are related to the scattering intensity.

In this article, the Jones calculus describes the experimental process but the numerical inferences are presented according to the Mueller calculus with respect to the depolarized system. Therefore, we translated the target signatures from the Jones calculus to the Mueller calculus, since the Mueller calculus describes the target signatures more reliably, as pointed out in [11].

## 3. Polarimetric Decomposition of Feature Space

This section is devoted to the baseline approaches that are used in the case of coherent decomposition. We will show the main advantages and disadvantages of these techniques with respect to wave propagation along a rough underlying surface.

Radar wave propagation leads to its scattering by heterogeneous objects. In particular, a polarized radar wave reflected from an object has a different polarization structure. One of the main problems for the scattered signal is its decomposition into an arbitrary orthogonal basis $\left(e_{i},e_{j}\right)=\delta_{ij}$, where $\delta_{ij}$ is the Kronecker delta. In this case, we can represent the scattering mechanism $S^{R}$ from Equation (2) in the canonical form $S^{R}=\sum_{k=1}^{N=3}\alpha_{k}S_{k}^{R}$, where $\alpha_{k},\;k\in 1\ldots 3$ are the decomposition coefficients. 

### 3.1. Pauli Decomposition

We consider the unitary group $SU\left(2\right)$ to decompose the scattering matrix in the canonical form $S^{R}$. The coherent matrix $T_{COH}\left\{3\times 3\right\}$ will be written in this group using the Hermitian matrix which is obtained from the Jones vector (Equation (1)):

(4) $$J\times J^{†}=\left(\begin{array}{cc} J_{X}J_{X}^{{*}^{}} & J_{X}J_{Y}^{{*}^{}}\\ J_{Y}J_{X}^{{*}^{}} & J_{Y}J_{Y}^{{*}^{}} \end{array}\right)$$

The resulting matrix can be represented as the sum of coefficients $S={\left[s_{0},\;s_{1},\;s_{2},\;s_{3}\right]}^{T}$ and basic functions $\left\{e_{a},e_{b},e_{c},e_{d}\right\}$ [5]:

(5) $$J\times J^{†}=s_{0}e_{a}+s_{1}e_{b}+s_{2}e_{c}+s_{3}e_{d}$$

According to the coherent decomposition theorem [14], linear combination (Equation (5)) can be written in the form of the following Pauli matrices:

(6) $$S_{P}=\frac{s_{0}}{\sqrt{2}}\left(\begin{array}{cc} 1 & 0\\ 0 & 1 \end{array}\right)+\frac{s_{1}}{\sqrt{2}}\left(\begin{array}{cc} 1 & 0\\ 0 & -1 \end{array}\right)+\frac{s_{2}}{\sqrt{2}}\left(\begin{array}{cc} 0 & 1\\ 1 & 0 \end{array}\right)+\frac{s_{3}}{\sqrt{2}}\left(\begin{array}{cc} 0 & -j\\ j & 0 \end{array}\right)$$

In the monostatic case, where $s_{3}=0$, the Pauli matrix basis can be reduced to the first three matrices. However, due to scattering on the underlying surface, it is necessary to assume $s_{3}\neq 0$.

### 3.2. Krogager Decomposition

The Krogager decomposition can be represented for linear and circular polarization, respectively:

(7a) $$S_{LIN}^{K}=e^{j\phi_{LIN}}k_{S}\left(\begin{array}{cc} 1 & 0\\ 0 & 1 \end{array}\right)+k_{D}\left(\begin{array}{cc} \cos2\theta & \sin2\theta \\ \sin2\theta & -\cos2\theta \end{array}\right)+k_{H}e^{\mp j2\theta }\left(\begin{array}{cc} 0 & \pm j\\ \pm j & 0 \end{array}\right)$$

(7b) $$S_{CIR}^{K}=e^{j\phi_{CIR}}k_{S}\left(\begin{array}{cc} 0 & j\\ j & 0 \end{array}\right)+k_{D}\left(\begin{array}{cc} e^{j2\theta } & 0\\ 0 & -e^{-j2\theta } \end{array}\right)+k_{H}\left(\begin{array}{cc} e^{j2\theta } & 0\\ 0 & 0 \end{array}\right)$$

where $k_{S}$, $k_{D}$, and $k_{H}$ denote corresponding to the sphere, diplane, and helix contribution, θ is the orientation angle, and $\phi_{LIN}=\phi_{HV}-0.5\left(\phi_{HH}-\phi_{VV}\right)\;\forall \phi_{LIN}\in S_{LIN}^{K}$ or $\phi_{CIR}=\phi_{RL}-0.5\left(\phi_{RR}-\phi_{LL}\right)\;\forall \phi_{CIR}\in S_{CIR}^{K}$, respectively. 

The Krogager decomposition demonstrates the real physical scattering mechanisms represented by the component matrices. It is obvious that scattering on non-spherical objects (cars and trucks) provides $k_{S}\to 1$. Furthermore, there is no orthogonality condition for the components between the sphere and “diplane–helix”, as stated in [32]. The decomposition elements are not basis-invariant. In addition, the phases $\phi_{LIN}$ and $\phi_{CIR}$ depend substantially on the scattering geometry on the underlying surface (see Section 4). Thus, the choice of the Krogager decomposition components as classifying features is inexpedient.

### 3.3. Cameron Decomposition

The Cameron approach (using the basis proportional to the Pauli matrices) [33] can be presented as

(8) $$S_{C}=\left[\cos\left(\psi_{rec}\right)\left[s_{\max}^{sym}\cos\left(\delta_{sym}\right)+s_{\min}^{sym}\sin\left(\delta_{sym}\right)\right]+\sin\left(\psi_{rec}\right)s^{nonrec}\right]$$

where $s_{\max}^{sym},\;s_{\min}^{sym}$ are the normalized maximum and minimum of symmetric components, $s^{nonrec}$ is the normalized nonreciprocal component, $\psi_{rec}$ is the reciprocity degree of the scattering matrix, and $\delta_{sym}$ is the deviation degree corresponding to symmetric scattering.

Two fundamental physical properties of radar scattering introduced by Cameron are reciprocity and symmetry. A scattering matrix S with $\theta_{rec}=0$ corresponds to a scatterer that strictly obeys the reciprocity principle, whereas a scattering matrix with $\theta_{rec}=\pi /2$ corresponds to a completely nonreciprocal scatterer.

In this case, Cameron establishes [34] that reciprocity is defined as $\theta_{rec}=\cos^{-1}\left\|P_{rec}S^{R}\right\|$, where $\left\|\cdot \right\|$ is the Euclidean norm; $P_{rec}\in {\mathbb{C}}^{4}$ is the projection operator chosen as

$$P_{rec}=\left(\begin{array}{cccc} 1 & 0 & 0 & 0\\ 0 & 1/2 & 1/2\; & 0\\ 0 & 1/2 & 1/2 & 0\\ 0 & 0 & 0 & 1 \end{array}\right),$$

where $S^{R}$ is the scattering vector from expression (2), which can be represented as $S^{R}=S_{rec}+S_{⊥}=S_{rec}+\left(I-P_{rec}\right)S^{R}$ with components of reciprocity $S_{rec}$, orthogonality $S_{⊥}$ and identity operator I.

When a wave is reflected from a rough underlying surface $S_{rec}\ll S_{⊥}$, the effects of additive and multiplicative interference can partially compensate for these losses (see Section 4). The second component in Expression (8) is the symmetry of the matrix $M\left\{4\times 4\right\}$. A symmetric scatterer is defined as a scatterer that has a symmetry axis in the plane orthogonal to the radar line of sight. Obviously, the symmetry is partially present with respect to the object under study: $\delta_{sym}\to \left\{0,\pi /2\right\}$. In the general case, the symmetry can disappear due to the influence of the underlying surface: $\delta_{sym}\to \pi /4$.

The feature space cannot be defined with reliable accuracy in all models (Pauli decomposition, Krogager decomposition, Cameron decomposition) [17,22]. The Pauli decomposition allows one to study the properties of these components as orthogonal elements on the Poincare sphere depending on the profile. A number of papers confirm the last thesis. First, the polarized scattering components can be represented as a linear combination of eigenvalues and eigenvectors [1,5,11]. The decomposition into eigenvectors and eigenvalues for the Mueller matrix can be an efficient approach to polarimetric recognition [34,35]. The recognition approach is to use the coherence matrix (Equation (2)) as the H/A/α polarimetric decomposition [11,14].

### 3.4. Assessment of Feature Space for the Learning Algorithm

Given the different features of coherent decomposition (see Section 3), in this section we consider the feature space for classifying ground vehicles. We also demonstrate criteria for evaluating the classification efficiency.

ATR of depolarized scattering is recommended to be carried out in the orthogonal basis according to principal component analysis [36]. Taking into account expression (5), the class of features can be described as follows:

$$X=X{\left(\begin{array}{ccc} x_{1}=s_{1}, & x_{2}=s_{2}, & x_{3}=s_{3} \end{array}\right)}^{T}.$$

Now we will develop the problem of classifying objects by polarization features. Let a learning sample $\left\{\left(x_{1},y_{1}\right),\ldots ,\left(x_{n},y_{n}\right)\right\}$ be given such that an arbitrary value of $x_{i}\in X$ uniquely corresponds to a known value of $y_{j}\in Y$. It is necessary to find a conversion function $a\left(x_{i},\;y_{i}\right):X\to Y$ that minimizes the loss function $L\left(Y\right)$ for a wide class of problems $L\left(Y\right):Y\times Y\to \mathbb{R}$. Hence, it satisfies the condition:

(9) $$L\left(Y\right)=\underset{y\in Y}{\arg\min}\left[a\left(y_{i}\right)\right]$$

An important task is to estimate $L\left(Y\right)$ as a result of binary classification. In order to obtain performance metrics, let us apply the classification sensitivity (TRP) to the polarization data, retrieved from the sample space

(10) $$TRP=\frac{TP}{TP+FN}$$

where TP is a true positive (type of vehicle correctly identified as a given type), FN is false negative (type of vehicle incorrectly identified as a given type).

Adoption of a hypothesis-testing approach corresponds to positive predictive value (PPV) and true positive rate (TPR), respectively. Therefore, we should apply the general characteristics (Equations (11) and (12)) to the binary classification. In general, these are accuracy measures of different tests:

(11) $$F_{1}=2\frac{TRP\times PPV}{TRP+PPV}$$

where F_1_ score is the harmonic mean value of precision and recall.

(12) $$\left|MCC\right|=\sqrt{\frac{\chi^{2}}{n}}$$

where MCC (Matthew’s correlation coefficient) is a metric as a measure of binary classification quality, $\chi^{2}$ is a chi-square statistic for a 2 × 2 table, and n is the total number of observations. Why should we use expressions (11) and (12)? Actually, the backscattering signal can produce an incorrect component from Equation (2). Hence, there would be confusion in classifying objects. We need an integral test for significance. 

## 4. Data and Calibration

The first task is to calibrate the radar according to the experimental conditions. It is necessary to exclude from consideration such phenomena as interference of direct and reflected waves, polarization mismatch, and spherical propagation of the radar signal. For this purpose, we used a stationary Ku-band radar with electronically scanned array (ESA), which generated a linear frequency-modulated waveform (LFMW). The station emitted and detected a polarized signal for the following mode:

- Linear polarization (vertical and horizontal plane)
- Circular polarization (right and left rotation).

The transmitted signal was scattered on the object of recognition located at the far end (~150 m). The radar receiver detected the reflected signal distorted by clutters. The signal value (in dB) was recorded on the radar display for each 10 ms. Unprocessed polarization marks were recorded in an open territory (a meadow) for direct visibility. For radar calibration, a fixed corner reflector was used in the far-field zone, also located at a distance of about 150 m.

The following objects were used for the experiment: a truck and a car. The profile of each object was used to estimate the scattering properties of the target. For this purpose, the object was rotated around its own axis by a full rotation. Registration of the signal was performed for each 15-degree displacement. The experimental conditions are given in Table 1.

Meadow unevenness (0.2–0.3 m) was taken into account as an average value of vegetation. During the entire observation period (about 2–3 h), a light wind prevailed, which influenced the fluctuations of the grass.

There are several approaches to calibration, such as singular decomposition or lexicographic decomposition, which are described in [37,38]. The calibration of our measurements was performed for a corner reflector [39,40]. It was assumed that under experimental conditions, there are additive and multiplicative interferences, which can be described by the expression:

(13) $$S_{R}=R^{N}S_{T}T^{N}+N$$

where $S_{R}$, $S_{T}$ are the 2 × 2 Jones scattering matrices according to Equation (1), $T^{N}$ is the transmitting distortion matrix corresponding to the multiplicative distortion component in the source-target direction, $R^{N}$ is the receiving distortion matrix corresponding to the multiplicative distortion component in the target-source direction, and N is the random additive distortion component of the ground surface. Our goal is to estimate the unknown values $R^{N}$, $T^{N}$, and N from Equation (13) in order to estimate the further corrected results for linearly polarized scattering (Section 4.1) and cyclically polarized scattering (Section 4.2).

**Figure 3:**
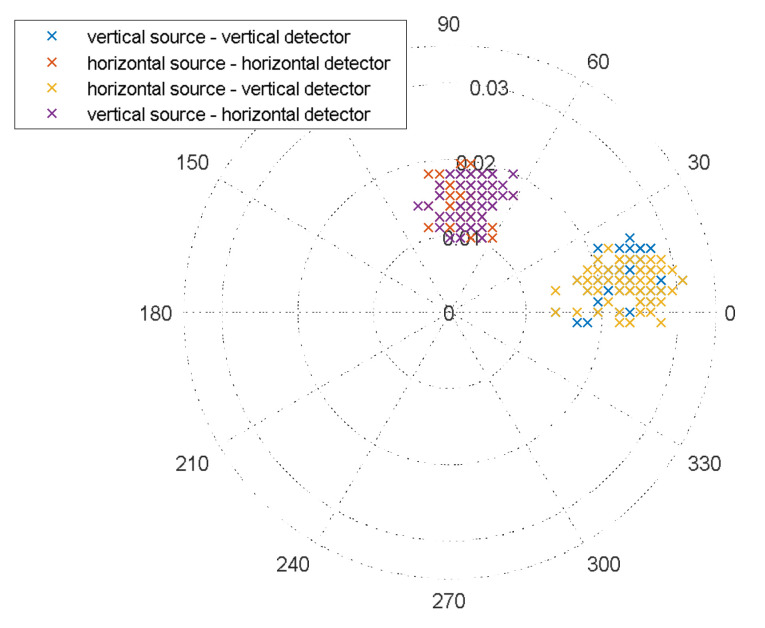
Polarization diagram of the received signal relative to the transmitted signal unit for a rough surface and free meadow propagation (radial axis is a dimensionless variable; linear polarization; number of marks for each observation—64).

In this work, the depolarizing properties of the rough surface (meadow) were taken into account as an additional component $\left(N\right)$. This was not done in [29,40], where studies were carried out under laboratory conditions. The target marks of the received scattered signal for free propagation and the influence of a rough terrain are shown in Figure 3 in polar coordinates.

Cross scattering prevails here: $\left|\varphi_{VV}-\varphi_{HH}\right|=\left|\varphi_{HV}-\varphi_{VH}\right|$. The multiplicative component of the clutters was calibrated using a corner reflector made for linear and circular polarization.

If we use expressions for linear phases (Equation (7a)), then $\phi_{LIN}=1.5\left(\phi_{HV}\right)-0.5\left(\phi_{VH}\right)$. Consequently, the multiplier (Equation (7a)) tends to 1 − $e^{j\phi_{LIN}}\to 1$, and the spherical component (Equation (7a)) is $k_{S}\leq 1$. In addition, the scattering with phases $\left\{\varphi_{VV},\varphi_{HH},\varphi_{HV},\varphi_{VH}\right\}$ does not allow us to apply the reciprocity principle denoted in Equation (8). Thus, the experimental calibration data (Equation (13)) indicate the complexity of practical application of the Krogager and Cameron decompositions.

### 4.1. Linear Polarization Data

**Figure 4:**
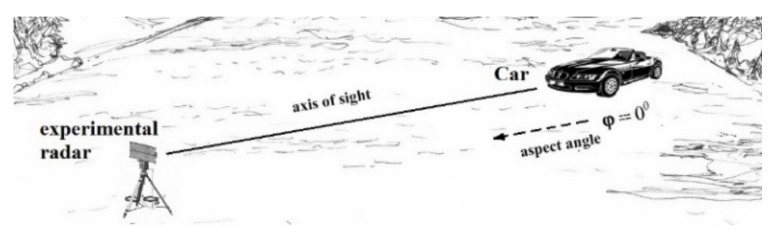
Scheme of the study.

Let us consider the case of linear polarization in scattering. The antenna pattern covers the object and the neighboring rough surface (Figure 4).

The synchronous detector was used at the output of the radar receiving path. It consisted of two channels: in-phase (I) and quadrature (Q), which made four types of linearly polarized signal: horizontal transmission and vertical reception IL1=ReHV, QL1=ImHV; vertical transmission and vertical reception IL2=ReVV, QL2=ImVV; vertical transmission and horizontal reception IL3=ReVH, QL3=ImVH; and horizontal transmission and horizontal reception IL4=ReHH, QL4=ImHH.

Let us consider a normalized transmitter wave. The Jones vector for the horizontal wave will be $E_{H}={\left[\begin{array}{cc} 1 & 0 \end{array}\right]}^{T}$ and for the vertical wave $E_{V}={\left[\begin{array}{cc} 0 & 1 \end{array}\right]}^{T}$. After combining both vectors, according to expression (1), we obtain:

(14) $$\left(\begin{array}{cc} I_{L4}+iQ_{L4} & I_{L1}+iQ_{L1}\\ I_{L3}+iQ_{L3} & I_{L2}+iQ_{L2} \end{array}\right)=\left(\begin{array}{cc} J_{11} & J_{12}\\ J_{21} & J_{22} \end{array}\right)\left(\begin{array}{cc} 1 & 0\\ 0 & 1 \end{array}\right)$$

**Figure 5:**
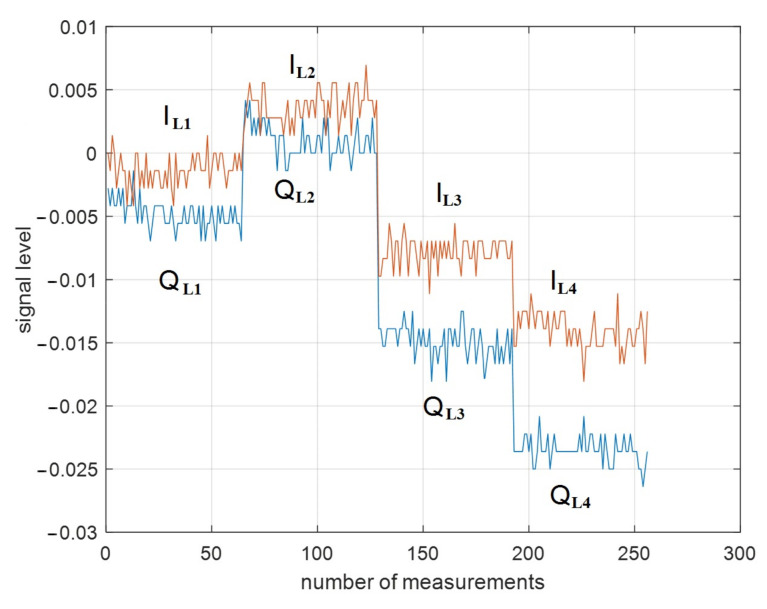
Unprocessed linear polarization target signature (truck, profile angle 45°): $I_{L1},\;Q_{L1}$—horizontal transmission and vertical reception, respectively; $I_{L2},\;Q_{L2}$ —vertical transmission and vertical reception, respectively; $I_{L3},\;Q_{L3}$ —vertical transmission and horizontal reception, respectively; $I_{L4},\;Q_{L4}$ —horizontal transmission and horizontal reception, respectively.

The detection of polarized scattering was carried out sequentially. Each polarization type contained 64 samples, after which a switch to another polarization mode occurred. An example of unprocessed target signature for linear polarization is shown in Figure 5.

The value of the detected signal, calculated as the average value of each component is $\bar{I}=\sum_{i}I_{i},\;\bar{Q}=\sum_{i}Q_{i}$. In addition, additive $\left(N_{LIN}\right)$ and multiplicative $\left(T_{LIN}^{N},\;R_{LIN}^{N}\right)$ linear scattering interferences, which are obtained after calibration of the measuring system, were taken into account. Then we expressed the Jones matrix through the obtained values according to (Equation (15)):

(15) $$J=R_{LIN}^{N}\left(\begin{array}{cc} {\bar{I}}_{L4}+{\bar{Q}}_{L4} & {\bar{I}}_{L1}+{\bar{Q}}_{L1}\\ {\bar{I}}_{L3}+{\bar{Q}}_{L3} & {\bar{I}}_{L2}+{\bar{Q}}_{L2} \end{array}\right)T_{LIN}^{N}+N_{LIN}$$

**Figure 6:**
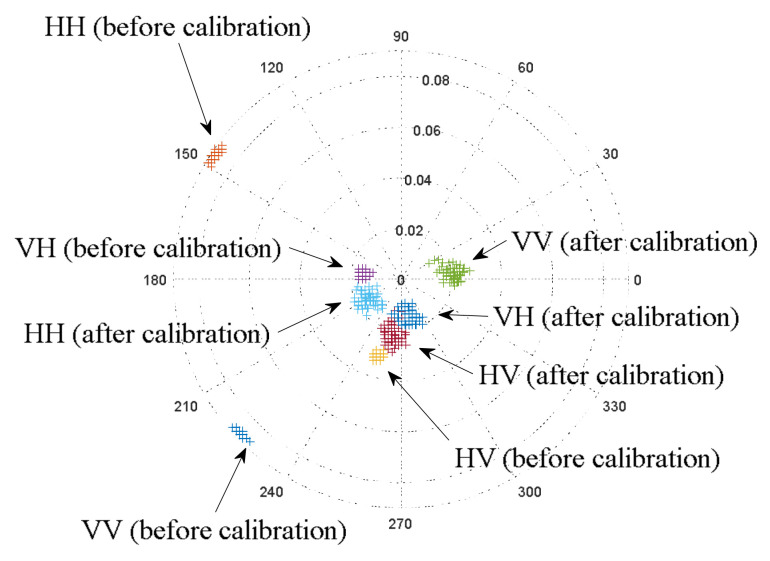
Target marks of the received signal (the second letter in the designation) relative to the transmitted signal unit (the first letter in the designation) in polar coordinates before and after calibration (linear polarization, car, profile angle 0°, number of marks for each observation—64).

Polarization marks of linear scattering before and after calibration are shown in Figure 6. The phase and amplitude displacements of the scattered signal relative to the transmitted signal with unit amplitude and corresponding zero phase are presented in the polar chart below.

After calibration, HV and VH are almost identical in the polar chart. The identical HV and VH responses confirm the reciprocal scattering [11]. Consequently, the calibration was performed correctly and the results make sense according to the physics. The experiment also showed that the obtained phase difference $\left|\varphi_{VV}-\varphi_{HH}\right|=8^{0}$ corresponds to the results stated in [1] (p. 59), where the mean square error is $\sigma =6.8^{0}$. Below is the polarimetric coherence matrix (Equation (2)) in the case of a car with zero profile angle (64 samples for each polarization channel):

(16) $$T_{4}=10^{-3}\times \left(\begin{array}{cccc} 0.6970 & 0.1673 & -0.0240 & 0.6498\\ -0.3308 & -0.0298 & 0.1649 & -0.3410\\ 0.1750 & 0.2163 & -0.0422 & 0.1304\\ -0.5575 & -0.1236 & -0.0810 & -0.5692 \end{array}\right)$$

The scattering matrix (Equation (16)) is dominated by the component $s_{3}$ of the main diagonal, except for the common component $s_{1}$. Obviously, this is due to the influence of vegetation fluctuations, since the component $s_{3}$ is the result of asynchronous scattering. We will confirm this statement in Section 6.1.

### 4.2. Circular Polarization Data

**Figure 7:**
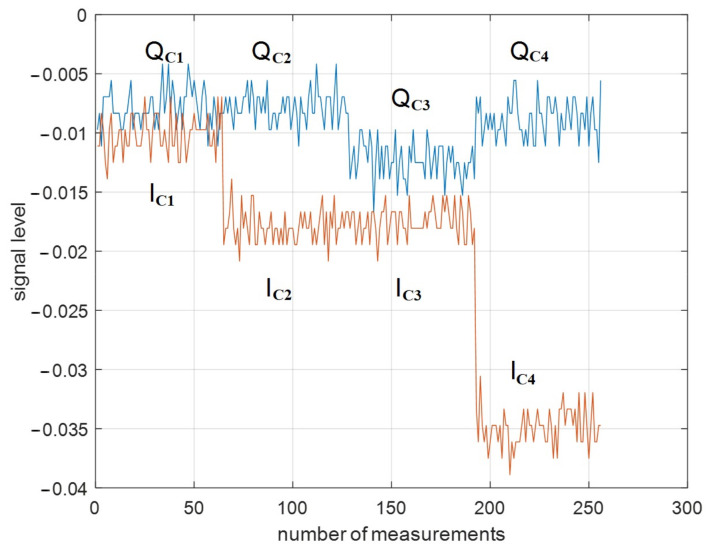
Unprocessed circularly polarized target signature of circular polarization (truck, profile angle 45°): $I_{C1},\;Q_{C1}$—left-hand circular transmission and right-hand circular reception, respectively; $I_{C2},\;Q_{C2}$ —right-hand circular transmission and right-hand circular reception, respectively; $I_{C3},\;Q_{C3}$ —right-hand transmission and left-hand circular reception, respectively; $I_{C4},\;Q_{C4}$ —left-hand circular transmission and left-hand circular reception, respectively.

Similar to linear polarization, a research experiment was performed for circular polarization (Figure 7).

The Jones vector will be $E_{LC}=1/\sqrt{2}{\left[\begin{array}{cc} 1 & i \end{array}\right]}^{T}$ for left-hand circular polarization and $E_{RC}=1/\sqrt{2}{\left[\begin{array}{cc} 1 & -i \end{array}\right]}^{T}$ for right-hand circular polarization. Elements of the Jones matrix can be calculated according to Equation (1):

(17) $$J=R_{CIR}^{N}\left(\begin{array}{cc} \left({\bar{I}}_{C3}+{\bar{I}}_{C4}\right)+\left({\bar{Q}}_{C3}+{\bar{Q}}_{C4}\right) & \left({\bar{I}}_{C3}-{\bar{I}}_{C4}\right)-\left({\bar{Q}}_{C3}-{\bar{Q}}_{C4}\right)\\ \left({\bar{I}}_{C1}+{\bar{I}}_{C2}\right)+\left({\bar{Q}}_{C1}+{\bar{Q}}_{C2}\right) & \left({\bar{I}}_{C1}-{\bar{I}}_{C2}\right)-\left({\bar{Q}}_{C1}-{\bar{Q}}_{C2}\right) \end{array}\right)T_{CIR}^{N}+N_{CIR}$$

where $T_{CIR}^{N}$, $R_{CIR}^{N}$ are the distortion matrices after calibration, and $N_{CIR}$ is the added matrix of interferences of circular scattering.

**Figure 8:**
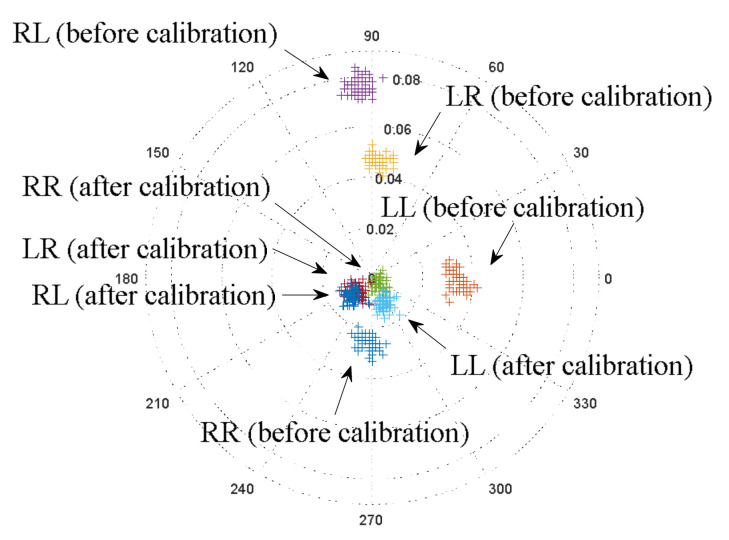
Target marks of the received wave relative to the normalized transmitted signal in polar coordinates before and after calibration (circular polarization, car, profile angle 0°, number of marks for each observation—64): LL—left-hand circular transmission and left-hand circular reception; RR—right-hand circular transmission and right-hand circular reception; RL—right-hand circular transmission and left-hand circular reception; LR is left-circular transmission and right-hand circular reception.

Polarization marks of circular scattering before and after calibration are shown in Figure 8.

The circular calibration demonstrates the reciprocity condition $\left|S_{RL}\right|\approx \left|S_{LR}\right|$. Obviously, this group has mutually correlated properties compared to linear polarization [31].

The averaged value of the polarimetric coherence matrix (car, profile angle 0°) is as follows:

(18) $$T_{4}=10^{-3}\times \left(\begin{array}{cccc} 0.1671 & 0.0362 & 0.0317 & -0.0834\\ 0.0785 & -0.0200 & 0.0377 & -0.1516\\ 0.0256 & 0.0360 & 0.1333 & 0.0151\\ 0.0496 & 0.1351 & -0.0214 & -0.0488 \end{array}\right)$$

The cross coefficients $m_{42}\approx 0.13$ and $m_{24}\approx -0.15$ are maximal in absolute value. Thus, in the case of a circular emitting wave, dihedral corner scattering prevails. The degree of polarization is DOP=0.8422. It is compatible with the results of linear polarization.

The ground surface effects for both types of target polarization signatures were removed using corner reflector measurements. The test readings were compared with additive and multiplicative components. It was found that the signal/noise ratio *SNR_LP_* = 6.8 for linear polarization and *SNR_EP_ =* 17.4 for circular polarization, respectively. Thus, the signal attenuation of the wave with linear polarization significantly exceeds the circular polarization attenuation (more than 10 dB).

## 5. Supervised Learning for Polarimetric Recognition

This section describes how features (see Section 3) can be used for supervised learning. It is important to emphasize that Bayesian inference [41] and artificial neural networks [17] are effective methods for classifying polarimetric data. A convolutional neural network [2,3,28,42] is used for unprocessed SAR images. Since we use supervised learning for a small volume of data, linear separability was chosen as the method of data analysis.

### 5.1. Modeling Polarimetric Recognition

**Figure 9:**
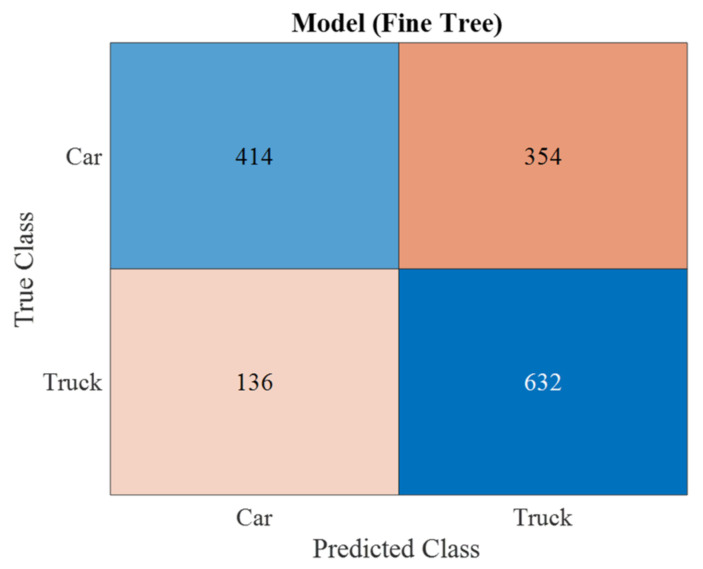
Confusion matrix for linear polarization (algorithm—fine tree (68%); split criterion—Gini’s diversity index).

**Figure 10:**
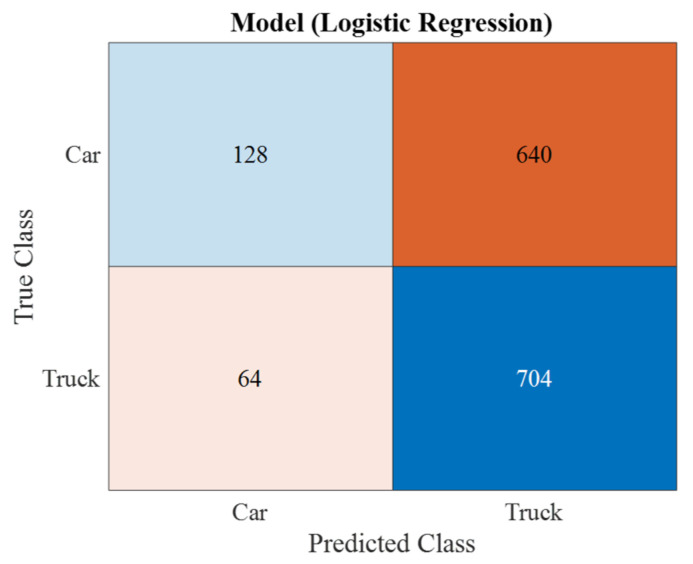
Confusion matrix for circular polarization (algorithm—logistic regression (54%); the hyper parameter option is disabled.

The values on the main diagonal of the $T_{4}$ matrix (16 and 18) were used as features for vehicle recognition and classification. Following expressions (15) and (17), we obtain true Pauli decomposition coefficients as if the radar signal propagated in free space without interference. Therefore, we need to choose the most significant coefficients. Coefficients exceeding the threshold level of radar sensitivity $P_{TH}>0.1$ were chosen. The results showed that vectors $S_{0}$, $S_{3}$ and $S_{0}$, $S_{2}$ satisfy the given conditions for linear polarization and circular polarization, respectively. Consequently, the featured vector consists of an array of data $X={\left[s_{0},\;s_{3}\left(s_{2}\right)\right]}^{T}$ obtained according to expressions (5) and (6). Class vectors contained two types of objects (car and truck). The observations consisted of 12 profile angles (from 0° to 165° in increments of 15°) in each class $Y={\left[{\left\{y_{i\_C}\right\}}_{i=0...165^{0}}^{k=12},\;{\left\{y_{i\_Tr}\right\}}_{i=0...165^{0}}^{k=12}\right]}^{T}$. That is, 64 target marks for each of the 12 profiles of a car or truck were obtained in a single time interval. The total sample amounted to $\delta \left\{2\;features;\;2\;classes\right\}=1536$ (Figure 9 and Figure 10).

Having obtained the data, it became possible to develop a supervised learning algorithm [42,43]. The results showed that the “Fine tree” algorithm with Gini diversity index separability criterion with 68.1% of correct results was more effective for linearly polarized wave (Figure 9).

The results of circular polarization modeling demonstrated the efficiency of the logistic regression algorithm, where the recognition accuracy was 54.2% (Figure 10).

It is obvious that polarimetric radar performing remote sensing using linearly polarized wave (68.1%) has higher accuracy of binary classification compared to circularly polarized wave (54.2%). These results can be explained by the more sensitive properties of the linearly polarized wave to external factors (object profile, weather conditions, etc.).

### 5.2. Comparison with Similar Methods

We made a comparison with other methods of polarization recognition. In most papers [1,2,44,45], the effect of a real rough surface on asymmetric scattering is missing.

In article [2], the emphasis is on HRRP technology, with 72% confusion in the class. Although the study was performed on 720 profiles, this does not imply verification of the finished results since the data sets included simulated samples.

In [44], it is proposed, using the nearest neighbor method, to classify objects. The accuracy of target recognition is proportional to the signal/noise ratio (validity reaches 82% at 50 dB). In [1], a convolutional neural network was made for seven classes of objects, where the average classification accuracy is 88%. Finally, after analyzing the model results for SAR [45], it was found that the recognition validity of polarized target signatures is no more than 42%. Thus, the obtained results are potentially more valid than the ones for similar systems.

## 6. Results and Discussion

In this section we demonstrate how HRRP modifies the components of the Pauli decomposition (see Section 3). We also analyze the effect of weathering and give an estimate for binary classification as a result of supervised learning (see Section 5).

### 6.1. Influence of Different Target Profiles and Weather Conditions

The structure of polarized scattering can vary significantly depending on the profile angle of the target and climatic conditions. A number of articles have paid attention to these factors [15,30]. The influence of the profile on the recognition efficiency has been examined with respect to two types of polarized backscattering. We will also demonstrate the validity of the results in accordance with the experimental data.

**Figure 11:**
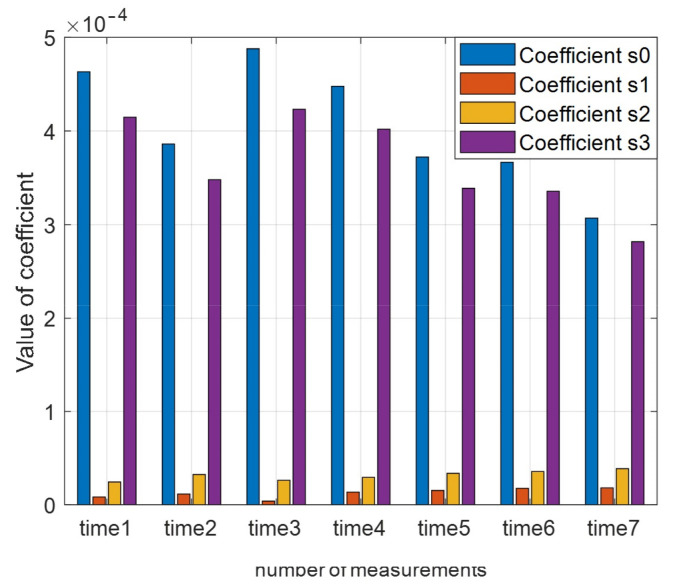
Scattering chart of a linear polarized wave from a trihedral reflector obtained at different time intervals during one day.

As a part of the study, we obtained the dependencies of coefficients S at different time intervals during one day. Obviously, it is necessary to take into account the fluctuations of grass in the meadow as an element of scattering. The histogram of the distribution $S={\left[s_{0},\;s_{1},\;s_{2},\;s_{3}\right]}^{T}$, constructed for a trihedral reflector in one profile, is shown in Figure 11.

It can be seen that the ratio of coefficients remains constant. At the same time, the intensity of the scattering fluctuations can reach 2.5 dB. Although the results obtained exceed the estimate for the road surface (>20 dB), as shown in [22], the value of asymmetric scattering in our case is much higher. For this reason, the feature space must consist of coefficients $S_{0}$ and $S_{3}$ for a rough real surface.

Appendix A shows an example of the distribution coefficients (truck) depending on the object profiles. If we consider a 90-degree object profile, the direct scattering ratio $\left|S_{VV}\right|$ exceeds the cross-polarized scattering $\left|S_{HV}\right|$ by 14.7 dB. Comparing this result with [46], where the value is 16.3 dB, we can confirm the data of our study.

According to Appendix A, there is no statistical pattern of modified profiles. Despite this result, the profile quantization step depends on the type of target recognition, as shown in [30], where a genetic algorithm for adaptive state selection of polarization angle radar sensing is analyzed. The general trend [1] shows that a very high angle quantization (no more than 1°) is required to construct a training sample. It is advisable to use convolutional technologies for this [2]. At the same time, since the asymmetric scattering coefficient $S_{3}$ dominates (Appendix A), it is possible to implement H/A/α polarimetric decomposition. However, such ATR analysis has often been performed for elevation angles greater than 30° [3,35,36]. There is reason to believe that ground type estimation is important to simplify the technique of polarimetric target recognition.

### 6.2. Estimation of Binary Classifiers

**Figure 12:**
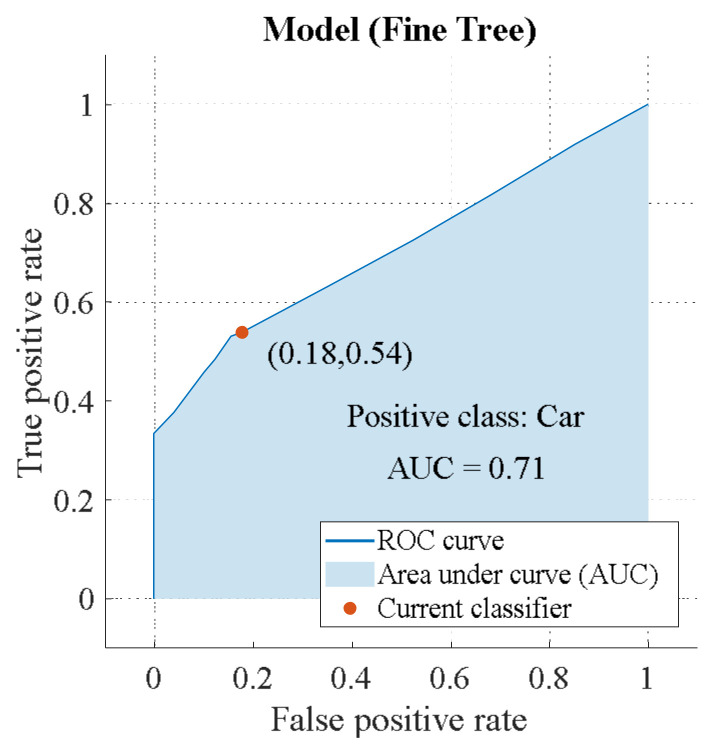
ROC of linear polarization (class “Car”).

**Figure 13:**
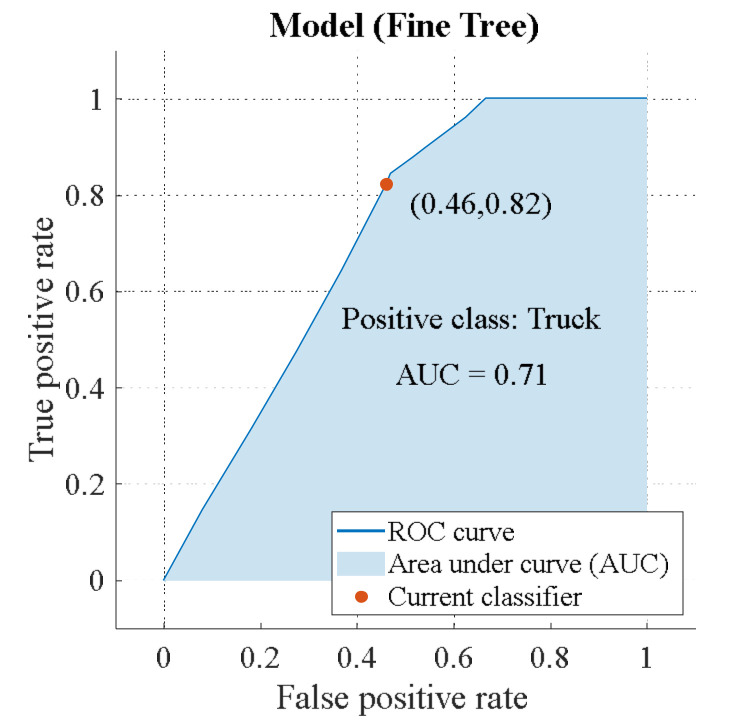
ROC of linear polarization (class “Truck”).

The accuracy of target recognition can be estimated using the receiver operating characteristic (ROC) curve. Then we get the efficiency of the binary classifier according to the area under the curve (AUC) value. Below is a chart showing the ROC of linear polarization for the classes “Car” and “Truck” (Figure 12 and Figure 13) [3].

**Figure 14:**
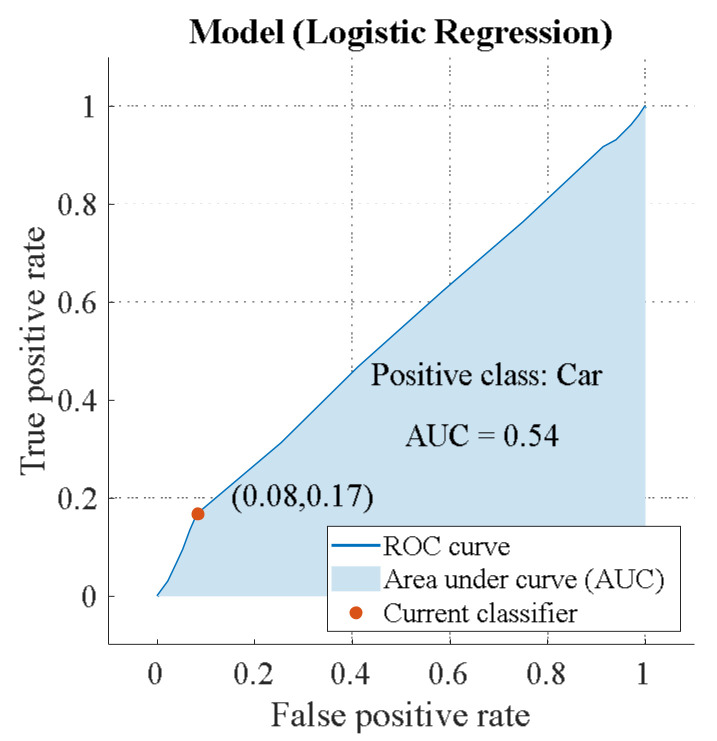
ROC of circular polarization (class “Car”).

**Figure 15:**
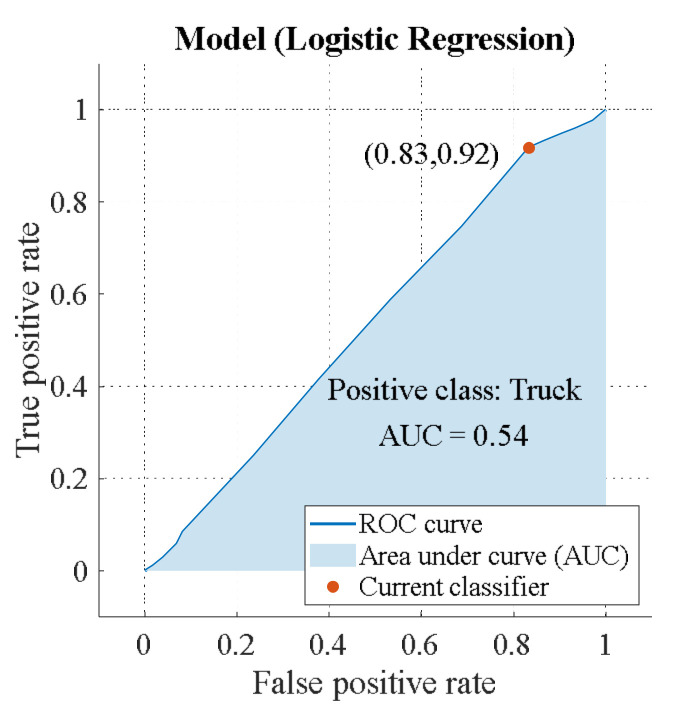
ROC of circular polarization (class “Truck”).

The recognition efficiency is the same for the “Car” and “Truck” classes: AUC = 0.71. However, there is a difference, which is demonstrated in Appendix B. “Truck” has a higher TRP = 82%, which allows the algorithm to detect this class correctly. “Car” has a lower FNP = 17%. Hence, the algorithm accurately rejects unwanted targets. Below is a circular polarization ROC (Figure 14 and Figure 15).

Potential efficiency of binary classification for a circular emitting wave $\left(AUC_{EL}=0.54\right)$ is lower than for a linearly polarized wave $\left(AUC_{LIN}=0.71\right)$. In addition, the classifier is able to detect “Truck” (TRP = 83%) more accurately than “Car” (TRP = 8%) (Appendix B).

Obviously, a linearly emitting polarized wave has significant advantages over a circular wave. At the same time, we need a general criterion capable of assessing not only the sensitivity of these classes (TPR), but also the classification accuracy (positive predictive value—PPV). Therefore, in this article we compared the accuracy of two tests, F1 score and Matthew’s correlation coefficient (Table 2).

The F-test showed that the “Truck” class has higher test accuracy than the “Car” class for the two polarization types. MCC shows the same results for both polarization types. The advantage of MCC over F1 is obvious, as these results correlate with the equality $AUC\left\{Car\right\}=AUC\left\{Truck\right\}$. Furthermore, MCC gives a more informative and truthful result when evaluating binary classifications than F1, according to [47,48]. As a result, we claim potentially higher confidence in the data for linearly polarized backscattering using the significance test.

## 7. Conclusions

The article demonstrates automatic target recognition—ATR (car and truck)—by polarimetric ground radar using the properties of polarized waves. Attention is paid to signal scattering, which occurs on rough terrain surfaces due to the strict geometry of wave propagation and vegetation fluctuation. Based on decomposition theorems in radar polarimetry, the article describes the degree of scattering of such processes. We analyzed various coherent decomposition approaches (Krogager, Cameron, and Pauli) and found that the Pauli decomposition is the most effective. An arbitrary vehicle has reflective properties, which can be estimated by the corresponding Pauli coefficients in the orthogonal basis of the scattering matrix. The feature space is chosen from two components of the Pauli decomposition, the threshold of which exceeds the desired value.

Significant variation of scattering coefficients depending on target profiles is a major problem in object recognition. This article demonstrates how to reduce additive and multiplicative clutters by calibrating measurements for a rough surface.

The Pauli coefficients obtained from experimental data of signal backscattering were applied in order to test the accuracy of recognition using famous algorithms of supervised learning. The most efficient algorithm of recognition turned out to be “Fine trees” (68.1% of correct answers for linear polarization). The logistic regression algorithm showed low accuracy for circular polarization (54.2%). According to Matthew’s correlation coefficient, linear scattering (0.3775) has a potential advantage over circular scattering (0.1260). Obviously, the properties of linear polarization can be used for object recognition. The circular scattering mechanism would not be recommended as a tool for object recognition on rough terrain surfaces.

## Figures and Tables

**Table 1:** Research conditions.

Parameter	Value
Distance to target	150 m
Sight angle	flat
Angles of profile	360° (periodicity 15°)
Radar resolution (range)	0.5 m
Radar resolution (azimuth)	1.8°
Time registration	10 ms

**Table 2:** Quality of binary classifications.

Type of Polarization	F1 Score	MCC
Linear (Car)	0.6282	0.3775
Linear (Truck)	0.7206	0.3775
Circular (Car)	0.2667	0.1260
Circular (Truck)	0.6667	0.1260

**Figure A1:**
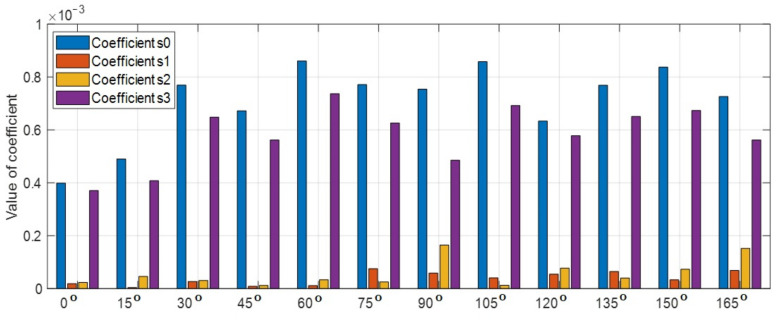
Linear coefficient data $S={\left[s_{0},\;s_{1},\;s_{2},\;s_{3}\right]}^{T}$ for truck: profile angle changes from 0° to 165° (periodicity 15°).

**Figure A2:**
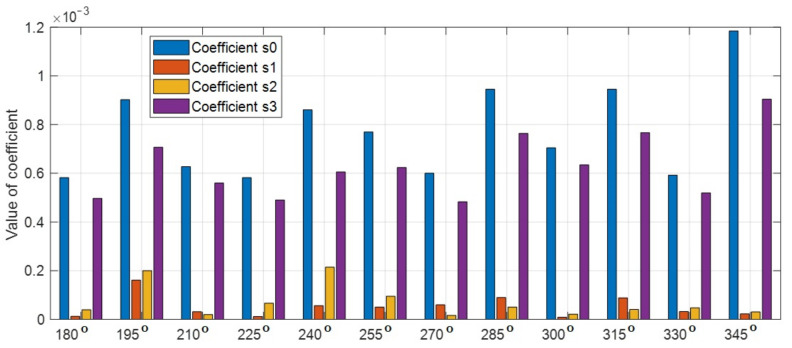
Linear coefficient data $S={\left[s_{0},\;s_{1},\;s_{2},\;s_{3}\right]}^{T}$ for truck: profile angle changes from 180° to 345° (periodicity 15°).

**Figure A3:**
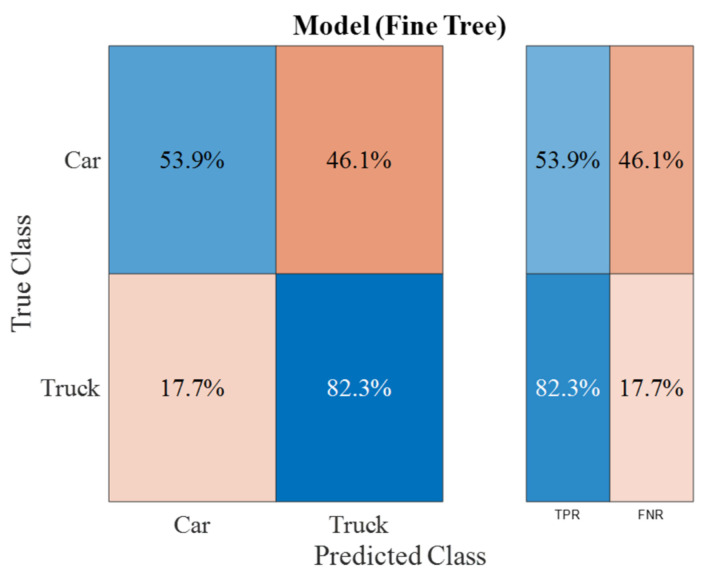
Confusion matrix TRP for linear polarization.

**Figure A4:**
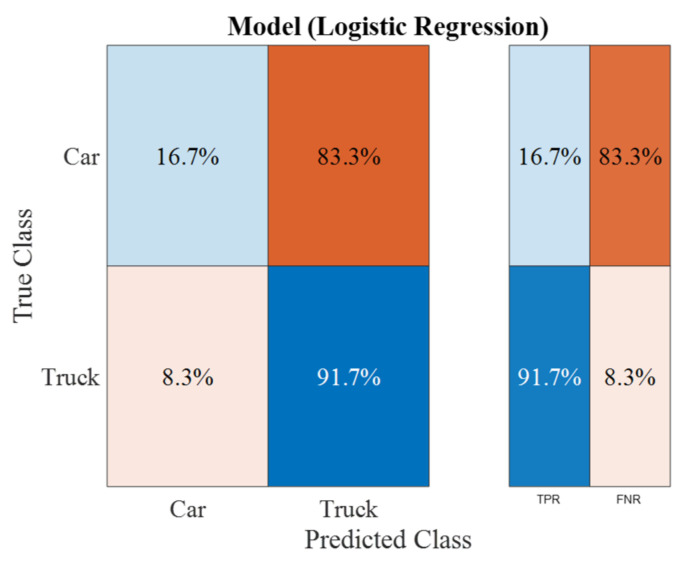
Confusion matrix TRP for circular polarization.

## References

[1] Visentin T. (2019). Polarimetric Radar for Automotive Applications. Ph.D. Thesis.

[2] He Y., Yang J. (2022). Polarization Estimation with a Single Vector Sensor for Radar Detection. Remote Sens..

[3] Yang F., Gao W., Xu B., Yang J. (2015). Multi-Frequency Polarimetric SAR Classification Based on Riemannian Manifold and Simultaneous Sparse Representation. Remote Sens..

[4] Li Y., Quan S., Xiang D., Wang W., Hu C., Liu Y., Wang X. (2020). Ship Recognition from Chaff Clouds with Sophisticated Polarimetric Decomposition. Remote Sens..

[5] Huynen J.R. (1970). Phenomenological Theory of Radar Targets. Ph.D. Thesis.

[6] Kennaugh E. (1952). Polarization Properties of Radar Reflection. Ph.D. Thesis.

[7] Mott H. (1986). Polarization in Antennas and Radar.

[8] Kvasnov A.V., Shkodyrev V.P., Arsenyev D.G. (2019). Method of recognition the radar emitting sources based on the naive bayesian classifier. WSEAS Trans. Systems Control.

[9] Cloude S.R., Pottier E. (1997). An entropy based classification scheme for land applications of polarimetric SAR. IEEE Trans. Geosci. Remote Sens..

[10] Cloude S.R., Pottier E. (1996). A review of target decomposition theorems in radar polarimetry. IEEE Trans. Geosci. Remote Sens..

[11] Lee J.S., Pottier E. (2009). Polarimetric Radar Imaging: From Basics to Applications. Optical Science and Engineering.

[12] Richards J.A. (2009). Remote Sensing with Imaging Radar.

[13] Kozlov A., Logvin A., Sarychev V., Shatrakov Y., Zavalishin I. (2020). Introduction to the Theory of Radiopolarimetric Navigation Systems.

[14] Cloude S.R. (2009). Polarization: Applications in Remote Sensing.

[15] Ryzhkov A.V., Zrnic D.S. (2019). Radar Polarimetry for Weather Observations.

[16] Rico-Ramirez M.A., Cluckie I.D. (2008). Classification of Ground Clutter and Anomalous Propagation Using Dual-Polarization Weather Radar. IEEE Trans. Geosci. Remote Sens..

[17] Da Silveira R.B., Holt A.R. (2001). An automatic identification of clutter and anomalous propagation in polarization-diversity weather radar data using neural networks. IEEE Trans. Geosci. Remote Sens..

[18] Torvik B., Olsen K.E., Griffiths H. (2016). Classification of Birds and UAVs Based on Radar Polarimetry. IEEE Geosci. Remote Sens. Lett..

[19] Zrnic D.S., Ryzhkov A.V. (1998). Observations of insects and birds with a polarimetric radar. IEEE Trans. Geosci. Remote Sens..

[20] Deep Y., Held P.P., Ram S., Dagmar Steinhauser D., Gupta A., Gruson F., Koch A., Roy A. (2020). Polarimetric Radar Cross-Sections of Pedestrians at Automotive Radar Frequencies. IET Radar, Sonar & Navigation. arXiv.

[21] Visentin T., Hasch J., Zwick T. Analysis of Multipath and DOA Detection Using a Fully Polarimetric Automotive Radar. Proceedings of the 2017 European Radar Conference (EURAD).

[22] Vassilev V. (2022). Road Surface Recognition at Mm-Wavelengths Using a Polarimetric Radar. IEEE Trans. Intell. Transp. Syst..

[23] Guo X., Yin J., Li K., Yang J., Shao Y. (2022). Scattering Intensity Analysis and Classification of Two Types of Rice Based on Multi-Temporal and Multi-Mode Simulated Compact Polarimetric SAR Data. Remote Sens..

[24] Sadjadi F. (2002). Improved target classification using optimum polarimetric SAR signatures. IEEE Trans. Aerosp. Electron. Syst..

[25] Zebker H.A., Van Zyl J.J. (1991). Imaging radar polarimetry: A review. Proc. IEEE.

[26] Shui P., Xu S., Liu H. (2011). Range-Spread Target Detection using Consecutive HRRPs. IEEE Trans. Aerosp. Electron. Syst..

[27] Jacobs S.P., O’Sullivan J.A. (2000). Automatic target recognition using sequences of high resolution radar range-profiles. IEEE Trans. Aerosp. Electron. Syst..

[28] Visentin T., Sagainov A., Hasch J., Zwick T. Classification of Objects in Polarimetric Radar Images Using CNNs at 77 GHz. Proceedings of the 2017 IEEE Asia Pacific Microwave Conference (APMC).

[29] Tatarinov V.N., Tatarinov S.V., van Genderen P. Principles of utilization of polarization invariant parameters for classification and recognition of distributed radar objects: Part I. Simplest model of a distributed object. Proceedings of the 2011 Tyrrhenian International Workshop on Digital Communications—Enhanced Surveillance of Aircraft and Vehicles.

[30] Sadjadi F. Technique for Selection of Optimum Polarimetric Angles in Radar Signature Classification. Proceedings of the IEEE International Radar Conference.

[31] Wang J., Liu Z., Ran L., Xie R. (2020). Feature Extraction Method for DCP HRRP-Based Radar Target Recognition via m−χ Decomposition and Sparsity-Preserving Discriminant Correlation Analysis. IEEE Sens. J..

[32] Touzi R. (2007). Target Scattering Decomposition in Terms of Roll-Invariant Target Parameters. IEEE Trans. Geosci. Remote Sens..

[33] Cameron W.L., Rais H. (2013). Polarization Scatter Feature Metric Space. IEEE Trans. Geosci. Remote Sens..

[34] Berizzi F., Martorella M., Capria A., Paladini R., Calugi D. H/α Polarimetric Features for Man-Made Target Classification. Proceedings of the 2008 IEEE Radar Conference.

[35] Long T., Zhang L., Li Y., Wang Y. (2019). Geometrical Structure Classification of Target HRRP Scattering Centers Based on Dual Polarimetric H/alpha Features. IEEE Access.

[36] Kawalec A., Owczarek R., Dudczyk J. Karhunen-Loeve Transformation in Radar Signal Features Processing. Proceedings of the 2006 International Conference on Microwaves, Radar & Wireless Communications.

[37] Lin J., Guo Y., Li W., Zhang Y., Chen Z. (2016). Polarimetric Calibration Based on Lexicographic-Basis Decomposition. IEEE Geosci. Remote Sens. Lett..

[38] Choudhary V., Rönnow D., Jansson M. A Singular Value Decomposition Based Approach for Classifying Concealed Objects in Short Range Polarimetric Radar Imaging. Proceedings of the 2019 PhotonIcs & Electromagnetics Research Symposium—Spring (PIERS-Spring).

[39] Wang S., Feng W., Sato M. Polarimetric Calibration for a Ground-based Synthetic Aperture Radar System. Proceedings of the 2019 Photonics & Electromagnetics Research Symposium—Fall (PIERS—Fall).

[40] Sarabandi K., Ulaby F.T., Tassoudji M.A. (1990). Calibration of polarimetric radar systems with good polarization isolation. IEEE Trans. Geosci. Remote Sens..

[41] Kvasnov A.V. (2020). Methodology of classification and recognition of the radar emission sources based on Bayesian programming. IET Radar Sonar Navig..

[42] Kvasnov A.V. Method of Classification of Fixed Ground Objects by Radar Images with the Use of Artificial Neural Networks. Proceedings of the International Conference on Cyber-Physical Systems and Control (CPS&C’2019).

[43] Kvasnov A.V., Shkodyrev V.P. (2021). A classification technique of civil objects by artificial neural networks using estimation of entropy on synthetic aperture radar images. J. Sens. Sens. Syst..

[44] Aldhubaib F., Shuley N.V. (2010). Radar Target Recognition Based on Modified Characteristic Polarization States. IEEE Trans. Aerosp. Electron. Syst..

[45] Jafari M., Maghsoudi Y., Valadan Zoej M.J. (2015). A New Method for Land Cover Characterization and Classification of Polarimetric SAR Data Using Polarimetric Signatures. IEEE J. Sel. Top. Appl. Earth Obs. Remote Sens..

[46] Visentin T., Hasch J., Zwick T. Polarimetric RCS Measurements of Selected Two-Wheeled Vehicles for Automotive Radar. Proceedings of the 2017 European Radar Conference (EURAD).

[47] Chicco D., Jurman G. (2020). The advantages of the Matthew’s correlation coefficient (MCC) over F1 score and accuracy in binary classification evaluation. BMC Genom..

[48] Kvasnov A.V., Nikitin N.A., Shkodyrev V.P. (2022). Experience in Design of Artificial Neural Network for Object Detection on Monochromatic Images. System Analysis in Engineering and Control.

